# Variation in access rates amongst patients groups in Wales for specialist interventions.

**DOI:** 10.23889/ijpds.v7i3.2081

**Published:** 2022-08-25

**Authors:** Jacqui Oakley, Robin Flaig, Emma Turner, Kirsteen Campbell, Katharine Evans, Stela McLachlan, Richard Thomas, Andrew Boyd

**Affiliations:** 1 Swansea University; 2 NHS Wales

## Objectives

The UK Longitudinal Linkage Collaboration (UKLLC) was established to link and integrate data from many longitudinal population studies (LPS) with participants’ records from diverse governmental data owners across the UK. The UKLLC aims to unify disparate requirements from many data owners in different legal jurisdictions into one, predictable, access framework.

## Approach

The UKLLC framework provides a legal and governance ‘funnel’ which enables diverse data owner requirements to be captured; for UKLLC to centrally control these; and - from a research applicants perspective - there to be a predictable single process that is bound into emerging national standards for data science (e.g., the ‘Five Safes’ framework). This is applied through a Trusted Research Environment (TRE) approach where ISO27001 and Digital Economy Act accredited contracts, data sharing agreements, policies and procedures define and permit data flows and provide organisational separation of key functions including the third-party processing of participant identifiers for linkages.

## Results

We developed a project review mechanism enabling UKLLC to efficiently process applications whilst respecting core data owners requirements: each aspect of this is tailored to relevant legal, jurisdictional and accreditation requirements. Contracting specialist processors enabled UKLLC to implement ‘organisational separation’ between use of participant identifiers and de-personalised data, enabling a ‘functionally anonymous’ approach facilitating efficient onward sharing of integrating data.

The processes we have developed promote transparency and trust with regulators, participants and public. UKLLC host (University of Bristol) is able to be the data controller of data within the UKLLC; make application decisions; create the Licenced Dataset; and, curate and reuse data and metadata products of the research whilst data is maintained functionally anonymous. Large-scale complex linked data-sets are provisioned in a timely manner.

## Conclusion

The UKLLC is part of a large ecosystem of organisations. A delegated and distributed model has been established supported by our contracts and policy. This configuration has allowed the delivery of cross-cutting data-sets to address priority Covid-19 research questions and a generalisable and sustainable model for the future

